# The Effect of Physical Activity Interventions Comprising Wearables and Smartphone Applications on Physical Activity: a Systematic Review and Meta-analysis

**DOI:** 10.1186/s40798-018-0157-9

**Published:** 2018-09-03

**Authors:** Roxanne Gal, Anne M. May, Elon J. van Overmeeren, Monique Simons, Evelyn M. Monninkhof

**Affiliations:** 1 Julius Center for Health Sciences and Primary Care, University Medical Center Utrecht, Utrecht University, STR 6.131, Universiteitsweg 100, PO Box 85500, 3508 GA Utrecht, The Netherlands; 2 Department of Human Geography and Spatial Planning, Utrecht University, Utrecht, The Netherlands

**Keywords:** Wearables, Smartphone applications, Physical activity

## Abstract

**Background:**

Worldwide physical activity levels of adults are declining, which is associated with increased chronic disease risk. Wearables and smartphone applications offer new opportunities to change physical activity behaviour. This systematic review summarizes the evidence regarding the effect of wearables and smartphone applications on promoting physical activity.

**Methods:**

PubMed, EMBASE and Cochrane databases were searched for RCTs, published since January 2008, on wearables and smartphone applications to promote physical activity. Studies were excluded when the study population consisted of children or adolescents, the intervention did not promote physical activity or comprised a minor part of the intervention, or the intervention was Internet-based and not accessible by smartphone. Risk of bias was assessed by the Cochrane collaboration tool. The primary outcome was changed in physical activity level. Meta-analyses were performed to assess the pooled effect on (moderate-to-vigorous) physical activity in minutes per day and daily step count.

**Results:**

Eighteen RCTs were included. Use of wearables and smartphone applications led to a small to moderate increase in physical activity in minutes per day (SMD = 0.43, 95% CI = 0.03 to 0.82; *I*^2^ = 85%) and a moderate increase in daily step count (SMD = 0.51, 95% CI = 0.12 to 0.91; *I*^2^ = 90%). When removing studies with an unclear or high risk of bias, intervention effects improved and statistical heterogeneity was removed.

**Conclusions:**

This meta-analysis showed a small to moderate effect of physical activity interventions comprising wearables and smartphone applications on physical activity. Hence, wearables and smartphone applications are likely to bring new opportunities in delivering tailored interventions to increase levels of physical activity.

## Key Points

- Interventions promoting physical activity may be enhanced if wearable devices, such as activity trackers, and smartphone applications are incorporated because effective behaviour change techniques can easily be integrated into wearables and smartphone applications.
- By exploring the factors influencing sustainability, adherence and long-term effectiveness, wearables and smartphone apps can be improved to increase effectiveness and optimize impact on public health.

## Background

Physical inactivity or low physical activity levels are an increasing problem worldwide [1–4]. Around 31% of the world’s population is classified as physically inactive, meaning that they are not meeting the physical activity (PA) recommendations [2]. Also, time spent in sedentary behaviour, defined as any waking behaviour while in a sitting, reclining or lying posture, is increased [4]. Physically inactive and sedentary people are at increased risk for non-communicable diseases such as cardiovascular disease, type 2 diabetes, some types of cancer and several other diseases and premature death [5–11]. It is estimated that inactivity is associated with a 20 to 30% increased mortality risk [8].

Increase in levels of physical activity holds the greatest potential to reduce premature death and to extend the lifespan [12]. High levels of moderate intensity physical activity (i.e. about 60 to 75 min per day) even seem to eliminate the increased risk of death associated with sedentary behaviour [13]. Therefore, stimulating physical activity gives potential for preventing a further increase in non-communicable diseases and premature death.

It is a challenge to reach physically inactive people and to promote and maintain physical activity behaviour change [14–16]. Several techniques are proposed for changing physical activity behaviour. For example, self-monitoring of behaviour is an important and effective technique [15–17], especially when combined with at least one of the following behavioural change techniques: prompt intention formation, prompt specific goal setting, provide feedback on performance and prompt review of behavioural goals [17, 18].

A meta-analysis including studies between January 2000 and August 2007 using pedometers showed a moderate positive effect on physical activity levels in adults and children. Compared to control groups, the intervention group increased on average by 2000 steps per day [19]. Another meta-analysis showed that Internet-delivered interventions, which are able to use different behavioural change techniques (e.g. providing information on consequences of behaviour, prompt barrier identification, relapse prevention and goal setting), were effective in producing small but significant increases in physical activity (*d* = 0.24, 95% CI = 0.09 to 0.38) [20, 21].

Advances in the device and smartphone technology, such as activity trackers and physical activity smartphone applications, have led to an exciting opportunity for delivering physical activity interventions [22]. In 2017, worldwide, there were over 2.3 billion smartphone users and more than 250,000 lifestyle apps available in the Google Play store [23, 24]. Sophisticated wearable devices (wearables) provide an easy and attractive way to self-monitor physical activity [25]. Likewise, advances in smartphone applications make it possible to use a combination of different behaviour change techniques to promote physical activity behaviour [23, 25, 26].

A previous systematic review from Coughlin et al. [27] showed promising results of smartphone apps in promoting physical activity, but the results were based on a combination of few randomized controlled trial and qualitative studies. Schoeppe et al. [28] found significant improvements in the physical activity of smartphone apps promoting physical activity in order to prevent non-communicable diseases in the intervention groups compared to the controls; however, no meta-analysis was performed. Studies included in these reviews were published between April 2007 and October 2014. Here, we summarize the findings of more recent randomized controlled trials and performed meta-analyses evaluating the effectiveness of physical activity interventions using wearables and smartphone applications to promote physical activity in adult populations compared to a control group.

## Methods

### Search Strategy

We conducted this review and reported according to the Preferred Reporting Items for Systematic Reviews and Meta-Analyses (PRISMA) guidelines [29]. This review was registered in the PROSPERO register of systematic reviews (CRD42015026529).

In August 2017, we searched on titles and abstracts in PubMed, EMBASE and the Cochrane Central Register of Controlled Trials (CENTRAL; 2008 to 2017). For the intervention, ‘mobile devices that promote physical activity’, we searched on the following MeSH terms or keywords: ‘Mobile applications OR Cell Phones OR Actigraphy’. We combined these with MeSH terms or keywords for the outcome ‘physical activity’: ‘Exercise OR Motor Activity’. We supplemented the keywords with searching in title/abstract using several synonyms of the intervention and outcome. The complete search strategy for all databases is available in the Appendix. We additionally searched the reference list of relevant reviews/studies.

### Study Selection

Studies were eligible for inclusion when they were randomized controlled trials (RCTs), conducted in adults, assessing wearables and/or smartphone- and/or tablet-based applications stimulating physical activity. Primary outcomes were time spent in (moderate-to-vigorous) physical activity, either objectively measured through pedometer or accelerometer data or subjectively measured by self-report questionnaires, and objectively measured daily step count.

Studies were excluded when the article was published before 2008 (introduction of the smartphone), the physical activity intervention comprised a minor part of a combined programme, or when the intervention was Internet-based and not accessible by smartphone. Control groups were excluded when they were offered the same application.

First, the title and abstract of the search yield were independently screened by four authors (RG, EM, EO, AM). Of potentially eligible studies, the definite selection was based on a full-text copy of the study, also independently screened by three authors (RG, EM, AM). Disagreement was resolved by consensus or by consulting a third author (EM or AM).

### Data Extraction

Three authors independently extracted the data from each of the included studies (RG, EM, EO). Discrepancies were resolved by consensus or consulting a third author (AM). We documented study characteristics (including year, author, study design), characteristics of the study population, components of the intervention (way of promoting physical activity, intervention duration) and outcomes on physical activity (including measures of physical activity, timing of measurements, statistical analysis, results). The five behaviour change techniques that were associated with the greatest effectiveness were documented as well [17, 18], that is, self-monitoring of physical activity, prompt intention formation (i.e. motivating to decide to act or set a goal, e.g. ‘I will take more exercise next week’), prompt specific goal setting (i.e. making a detailed planning of what to do), review of behavioural goals (i.e. reviewing and reconsidering previously set goals) and feedback on performance.

When a trial included more intervention arms, the intervention arm using a wearable and smartphone application combined with the fewest additional intervention components was compared with the control arm. Supplementary material or the website of the study, wearable or smartphone application was consulted when characteristics of the intervention were not described sufficiently. In case of missing data, we contacted the corresponding authors.

### Risk of Bias Assessment

The risk of bias of each included study was assessed by three independent authors (RG, EM, EO). We used the Cochrane risk of bias tool, consisting of six domains [30]. Each domain was scored as low, unclear or high risk of bias. Disagreement about the risk of bias assessments was resolved by consensus or consulting the third author. An overall classification of low, unclear or high risk of bias in each study was based on the combination of the domains.

The following domains were assessed:

- Sequence generation: Was the method used to generate the allocation sequence appropriate to produce comparable groups? If the method was not described, the risk of bias was rated as unclear.
- Allocation sequence concealment: Was the method used to conceal the allocation sequence appropriate to prevent the allocation being known in advance of, or during, enrolment? If the method was not described, the risk of bias was rated as unclear.
- Blinding of outcome assessment: How subjective or objective was the outcome assessment? Objectively measured physical activity outcomes were rated as low risk of bias, and subjective outcomes were rated as high risk of bias. Unblinded physical activity assessments were less likely to be biased when objectively measured.
- Incomplete outcome data: Were incomplete outcome data adequately addressed? Were attrition (drop-out) and exclusions from the analysis reported? Was the analysis an intention-to-treat analysis or were missing data imputed appropriately?
- Selective outcome reporting: Were outcomes prespecified in a study protocol or trial registration and reported as specified? If outcomes were not prespecified elsewhere, the risk of bias was rated as unclear.
- Other potential threats to validity: When baseline differences between study groups were present, were these accounted for in the analysis? Were there other sources of bias, not previously mentioned?

We decided to exclude the item blinding of participants because this is not feasible in these types of studies. However, we were aware that this shortcoming can lead to performance bias. The risk of bias assessment for blinding of outcome assessment was based on the method of outcome assessment (objective or subjective) and is already taken into account in the meta-analyses.

### Publication Bias

To investigate publication bias, we assessed funnel plots by visual inspection for asymmetry. In a funnel plot, the treatment effect is plotted against a measure of precision. When a publication is less likely for smaller and hence less precise studies failing to detect a significant effect, the funnel plot may be asymmetrical.

### Statistical Analyses

The primary outcome was time spent in (moderate-to-vigorous) physical activity in minutes per day and mean number of steps per day. Outcomes in minutes per week were converted to minutes per day divided by 7. When change scores from baseline to post-intervention were not available, outcome scores post-intervention were described. Intervention effects were assessed by comparing the difference in physical activity level between the intervention and control group. We used random-effects models, grouped by the method of assessing physical activity (objectively or subjectively) and type of outcome (moderate-to-vigorous) physical activity or daily step count [31]. Standardized mean differences (SMD) with a 95% confidence interval were calculated since different measurement instruments were used (Review Manager (RevMan), version 5.3) [32]. A standardized mean difference of 0.2 represents a small effect, 0.5 a moderate effect and 0.8 a large effect [33]. Statistical heterogeneity was assessed by examining the forest plots and calculating *I*^2^. The *I*^2^ statistic described the percentage of variability across studies that is due to heterogeneity rather than chance [34]. *I*^2^ values of 25, 50 and 75 were upper limits for low, moderate and high heterogeneity, respectively. Studies reporting multiple outcomes (e.g. objectively as well as subjectively measured) could be included in more than one meta-analysis.

Subgroup analysis was performed to explore clinical heterogeneity and subgroup effects, i.e. healthy versus diseased study populations and shorter (than 14 weeks) versus longer (than 14 weeks) intervention duration. Additional analyses were performed to test the robustness of the results by removing studies with an unclear or high risk of bias.

## Results

**Fig. 1:**
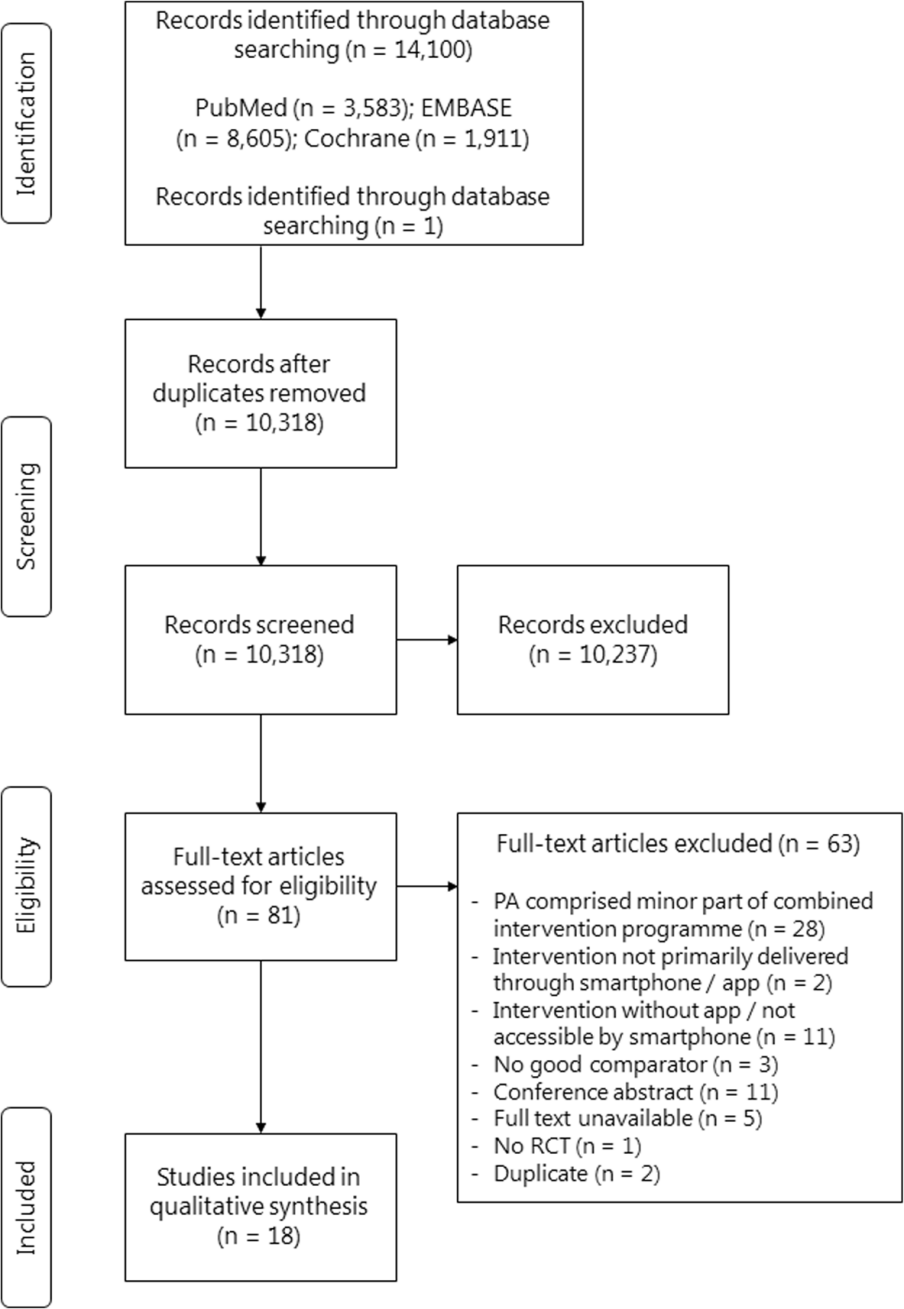
Flow diagram of trial selection, adapted from PRISMA. *PA* physical activity, *RCT* randomized controlled trial

Of the 10,318 unique articles screened on title and abstract, 81 full-text articles were assessed for eligibility (see Fig. 1). Finally, 18 studies were included in this review [35–52].

### Population and Intervention Characteristics

Table 1 presents the characteristics of the included studies and interventions. The included studies involved 2734 participants from different populations. Twelve studies included healthy adults, also including inactive and/or overweight [35, 36, 38–42, 44, 47–49, 52], whereas four studies were conducted in patients with (chronic) diseases [37, 43, 50, 51], one study in participants with an elevated risk of cardiovascular disease [45] and another study included stroke survivors [46]. In all studies, except one, physical activity was promoted through a smartphone or tablet application. The application was, in most studies, supported by a pedometer [36–39, 50, 52] or accelerometer [40–42, 44–47, 49, 51]. In the other study, physical activity was promoted by an accelerometer which could be synchronized with an online dashboard [43].

**Table 1:** Characteristics of included studies

Study	Population	*N* (randomized)/mean age (years)/sex (% female)	Intervention	Behaviour change techniques	Duration of intervention	Control
Li et al. [43]	Patients with knee osteoarthritis, > 50 years of age	34/55.5/82	Individualized PA goals Group education session and weekly telephone counselling by a physiotherapist Fitbit Flex activity tracker synchronized with an online dashboard	Self-monitoring Intention formation Specific goal setting Feedback on performance Review of behaviour goals	1 month	Waiting list (same intervention with 1-month delay)
Lyons et al. [44]	Overweight, inactive adults, aged 55–79 years	40/61.5/85	Daily and weekly step goals Instruction visit and weekly telephone counselling Jawbone Up24 and jawbone UP app	Self-monitoring Intention formation Specific goal setting Feedback on performance Review of behaviour goals	12 weeks	Waiting list (full intervention after final assessment)
Bickmore et al. [36]	Community-dwelling inactive adults, ≥ 65 years of age	263/71.3/61	Individualized short- and long-term PA goals Wearing pedometer ECA (computer-animated virtual exercise coach) on a tablet for 2 months ECA access in clinic waiting room for the following 10 months	Self-monitoring Intention formation Specific goal setting Feedback on performance Review of behaviour goals	12 months (2 months intervention phase + 10 months maintenance phase)	Wearing pedometer
Allen et al. [35]	Overweight adults, aged 21–65 years	34/44.9/78	PA goal of ≥ 150 min/week of MVPA Intensive counselling Lose it! weight loss application	Self-monitoring Intention formation Specific goal setting Feedback on performance Review of behaviour goals	6 months	Intensive counselling
Demeyer et al. [37]	COPD patients, > 40 years of age, smoking history ≥ 10 pack-years, stable or having an exacerbation in the last month	343/67/36	Individualized daily PA (step) goals Introduction visit, brochure and booklet containing home exercises Weekly group text message Fitbug Air step counter with Fitbug application and a project-tailored Proactive Linkcare coaching application	Self-monitoring Intention formation Specific goal setting Feedback on performance Review of behaviour goals	12 weeks	Usual care (standard leaflet explaining the importance of PA, information about PA recommendations)
Shin et al. [49]	Overweight Korean male university students, aged 19–45 years	70/27.8/0	Individualized PA goal to lose weight Brief education sessions on diet and exercise and education materials Fitmeter accelerometer and accompanying smartphone application (customized for the intervention)	Self-monitoring Intention formation Specific goal setting	12 weeks	Brief education sessions on diet and exercise and education materials
Recio-Rodriguez et al. [47]	Adults, ≤ 70 years of age, from Spanish primary care centres	833/51.9/62	Standardized counselling in PA and the Mediterranean diet EVIDENT II mobile phone app (designed for this study)	Self-monitoring Intention formation Specific goal setting Feedback on performance Review of behaviour goals	3 months	Standardized counselling
Uhm et al. [50]	Patients, aged 20–70 years, who completed primary breast cancer treatment	356/50.3/100	PA goal of ≥ 150 min/week of MVPA Home-based exercise programme Smart After Care exercise application and pedometer	Self-monitoring Intention formation Feedback on performance	12 weeks	PA goal of ≥ 150 min/week of MVPA Home-based exercise programme Exercise brochure
Glynn et al. [39]	Primary care patients	90/44.1/64	PA goal of 10,000 steps/day Accupedo Pro Pedometer App	Self-monitoring Intention formation Specific goal setting Feedback on performance Review of behaviour goals	8 weeks	PA goal of 30 min walking/day
Fukuoka et al. [38]	Overweight, inactive adults, ≥ 35 years of age, at risk for diabetes	61/55.2/77	Long-term PA goal of 12,000 steps/day In-person sessions Pedometer and Mobile phone-based Diabetes Prevention Program (mDPP) mobile app	Self-monitoring Intention formation Specific goal setting Feedback on performance Review of behaviour goals	5 months	Wore pedometer, displaying step count
Safran Naimark et al. [48]	Healthy adults interested in a healthy lifestyle	99/47.9/64	Presentation on healthy lifestyle eBalance application to promote a healthy lifestyle	Self-monitoring Intention formation Feedback on performance	14 weeks	Presentation on a healthy lifestyle
Martin et al. [45]	Adults visiting a cardiovascular disease prevention centre	32/58.0/46	PA goal of 10,000 steps/day First phase of unblinded digital activity tracking with Fitbug app Second phase with three automated, personalized, smartphone-delivered coaching messages per day	Self-monitoring Intention formation Specific goal setting Feedback on performance Review of behaviour goals	4 weeks (2 weeks phase 1 and 2 weeks phase 2)	Blinded digital activity tracking
Walsh et al. [52]	Young adults, aged 17–26 years, using a mobile phone	58/20.5/73	PA goal of 10,000 steps/day Information regarding the benefits of exercise Accupedo-Pro Pedometer App and accompanying pedometer	Self-monitoring Intention formation Specific goal setting Feedback on performance Review of behaviour goals	5 weeks	Education regarding PA goal of 30 min walking/day and benefits
Hartman et al. [41]	Middle-aged and older overweight women with elevated breast cancer risk	55/59.5/100	Weight loss intervention with MyFitnessPal and Fitbit app PA goal of ≥ 150 min/week of MVPA Twelve standardized coaching calls	Self-monitoring Intention formation Specific goal setting Feedback on performance Review of behaviour goals	6 months	Usual care (dietary guidelines) Two brief calls
Harries et al. [40]	Healthy males, aged 22–40 years	110/NA/0	Smartphone app recording steps and providing feedback Motivational text messages	Self-monitoring Intention formation Feedback on performance	6 weeks (plus 2 weeks run-in)	Carries smartphone to record steps with a built-in accelerometer
Vorrink et al. [51]	Patients with COPD, GOLD stage 2 or 3, ≥ 40 years of age	183/63.0/50	Individualized PA goal Smartphone application and built-in accelerometer Website for physiotherapist to monitor patient and provide feedback	Self-monitoring Intention formation Specific goal setting Feedback on performance Review of behaviour goals	12 months	Usual care
King et al. [42]	Underactive adults, ≥ 45 years of age, with no prior smartphone experience	49/60.0/75	Daily PA goal of 30 min/day of MVPA Smartphone app recording steps and providing feedback	Self-monitoring Intention formation Specific goal setting Feedback on performance Review of behaviour goals	8 weeks	Non-physical, diet-tracking control app (Calorific)
Paul et al. [46]	Stroke survivors	24/56.0/52	Individualized PA step goals, increased during the intervention period STARFISH smartphone app Extra research centre visit	Self-monitoring Specific goal setting Feedback on performance Review of behaviour goals	6 weeks	Usual care (wore ActivPAL before start intervention and during last week)

*COPD* chronic obstructive pulmonary disease, *ECA* computer-animated virtual exercise coach, *GOLD* Global Initiative for Chronic Obstructive Lung Disease, *MVPA* moderate-to-vigorous physical activity, *PA* physical activity

In all studies, the application included self-monitoring as a behaviour change technique [35–52]. Also, in all studies except one [40], goal setting was included. Approximately two thirds of the studies used setting of individualized physical activity goals as a behaviour change technique [36–38, 43, 44, 46–51], whereas the other studies used general goals (e.g. at least 150 min physical activity of at least moderate intensity or 10,000 steps per day) [35, 39, 41, 42, 45, 52]. In almost all studies except four [40, 48–50], reviewing and reconsidering previously set behavioural goals was included in the application. Furthermore, all studies except one [46] included prompt intention formation, and all studies included feedback on the performance except for one study [49].

Other intervention components in addition to the application were among others an introduction through a presentation, booklet or education visit [37, 44, 48–50] and counselling, in-person [35, 38, 47], by telephone [41, 43] or in a group session [43]. The duration of the intervention ranged from 4 weeks to 12 months. Control groups differed across studies, varying from usual care [37, 41, 42, 45, 46, 51] and waiting list [43, 44] to some form of education through a presentation, booklet or education visit [39, 48–51].

### Measurement of Physical Activity

Table 2 gives an overview of the measurement of the outcome (i.e. level of physical activity). Most studies measured physical activity objectively with an external accelerometer [37, 41, 43–47, 51] or pedometer [36, 38] or with a smartphone’s inbuilt accelerometer [40, 42] or pedometer [39, 52]. Four studies subjectively measured physical activity using a questionnaire [35, 48–50]. Most studies reported their results in mean minutes of physical activity per day [37, 38, 41–45, 47] or daily step count [37–40, 44–47, 51, 52]. Other reported outcomes were mean hours of physical activity per week [35], mean minutes of physical activity per week [48], kilocalorie per day [49] and metabolic equivalent of task (MET) per day [50]. Eleven studies reported a change in physical activity level between the baseline and the end of the intervention [35, 37–39, 41, 45, 47–49, 51, 52], and seven studies reported post-treatment physical activity level [36, 38, 41, 43, 44, 46, 50].

**Table 2:** Measurement of physical activity outcome

Study	Physical activity outcome	Outcome measurement instrument	Timing of measurement	Objective/subjective
Li et al. [43]	MVPA, ≥ 4 METs (min/day)	SenseWear Mini armband (research-based accelerometer)	Baseline and 1 month	Objective
Lyons et al. [44]	Stepping time (min/day) Steps/day	ActivPAL activity monitor	Baseline and 12 weeks (and 6 weeks)	Objective
Bickmore et al. [36]	Steps/day	Digital pedometer	12 months (and 2 months)	Objective
Allen et al. [35]	MVPA (hours/week)	Stanford 7-day Physical Activity Recall (PAR) questionnaire	Baseline and 6 months	Subjective
Demeyer et al. [37]	MPA (min/day) Steps/day	ActiGraph GT3X+ accelerometer	Baseline and 12 weeks	Objective
Shin et al. [49]	Kcal/day	International Physical Activity Questionnaire-Short Form (IPAQ-SF)	Baseline and 12 weeks	Subjective
Recio-Rodriguez et al. [47]	MVPA (min/week) Steps/day	ActiGraph GT3X accelerometer	Baseline and 3 months	Objective
Uhm et al. [50]	MET/week	International Physical Activity Questionnaire-Short Form (IPAQ-SF)	Baseline and 12 weeks	Subjective
Glynn et al. [39]	Steps/day	Accupedo Pro Pedometer App	Baseline and week 8 (and week 2)	Objective
Fukuoka et al. [38]	MPA (min/day) Steps/day	Omron Active Style Pro HJA-350IT pedometer	Baseline and 5 months (and every month)	Objective
Safran Naimark et al. [48]	PA (min/week)	Questionnaire-based on the International Physical Activity Questionnaire (IPAQ)	Baseline and 14 weeks	Subjective
Martin et al. [45]	MVPA (min/day) Steps/day	Fitbug Orb accelerometer	Baseline and 5 weeks	Objective
Walsh et al. [52]	Steps/day	Accupedo-Pro Pedometer App	Baseline and 5 weeks	Objective
Hartman et al. [41]	MVPA (min/day)	ActiGraph GT3X+ accelerometer	Baseline (week before randomization) and 6 months	Objective
Harries et al. [40]	Steps/day	Smartphone app bActive with a built-in accelerometer	Continuously during the trial. Mean number of steps in the 6th week is used.	Objective
Vorrink et al. [51]	Steps/day	SenseWear Mini armband (research-based accelerometer)	Baseline and 12 months (and 3 months and 6 months)	Objective
King et al. [42]	MVPA (min/day)	Smartphone-based accelerometer	Unknown	Objective
Paul et al. (2016) [46]	Steps/day	ActivPAL™ activity monitor	Baseline (7 days before the start of the intervention) and the last 7 days of the intervention period	Objective

*Kcal* kilocalorie, *MET* metabolic equivalent of task, *min* minutes, *MPA* moderate physical activity, *MVPA* moderate-to-vigorous physical activity, *PA* physical activity

### Risk of Bias of Included Studies

**Fig. 2:**
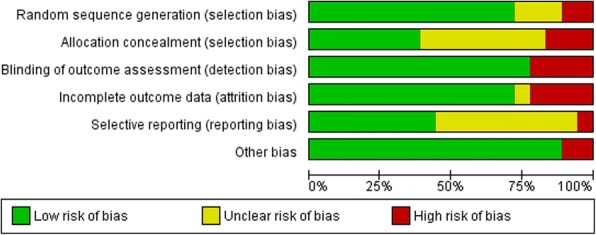
Risk of bias graph: review authors’ judgements about each risk of bias item presented as percentages across all included studies

**Fig. 3:**
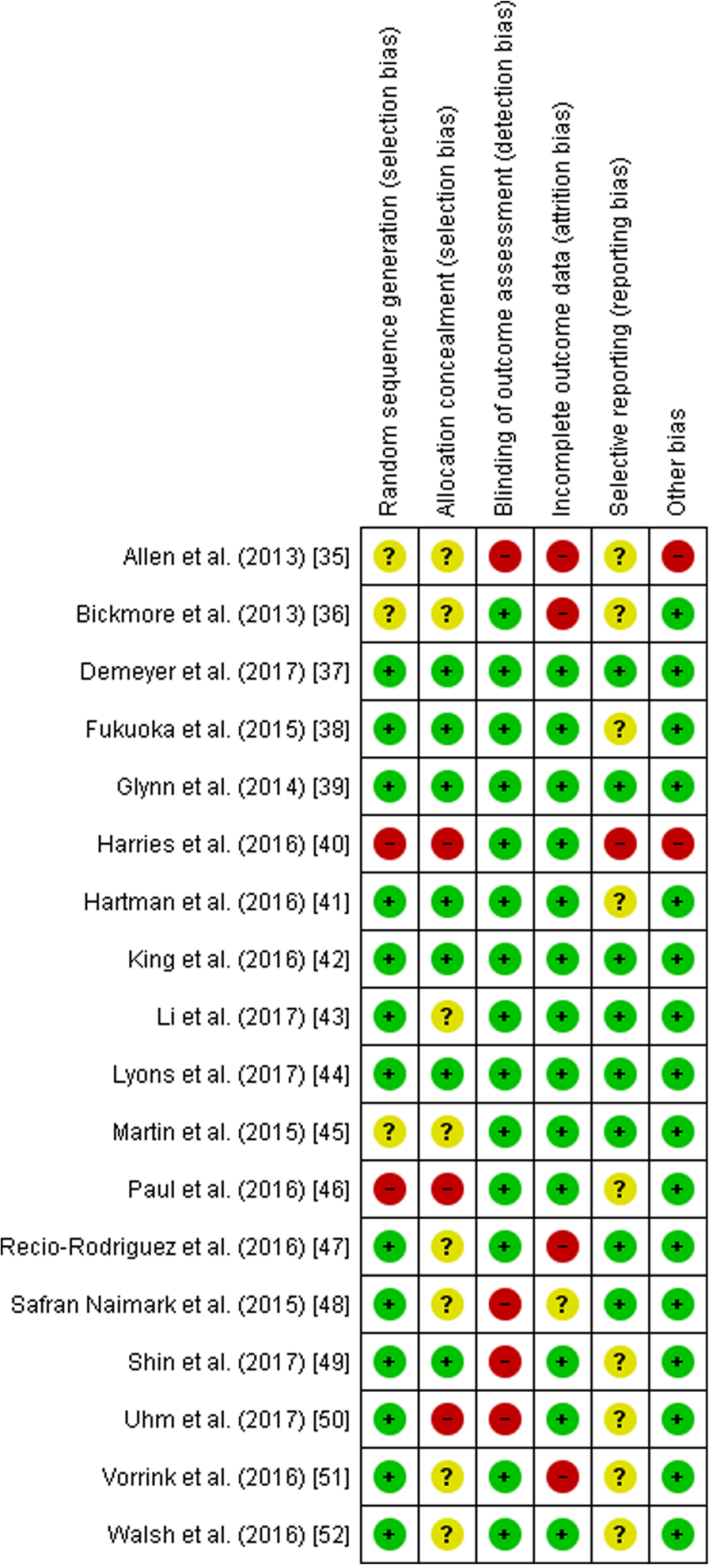
Risk of bias summary: review authors’ judgements about each risk of bias item for each included study. Green symbols represent a low risk of bias, yellow symbols represent an unclear risk of bias and the red symbols indicate a high risk of bias

Figures 2 and 3 show the risk of bias assessment of the 18 included studies. Three studies did not describe the method of randomization and were rated as an unclear risk of bias [35, 36, 45]. Two studies were at high risk of selection bias caused by the method of sequence generation. Harries et al. [40] listed participants in the order of recruitment, and each third participant was allocated to one of the three groups. Paul et al. [46] assigned the first eight participants to the intervention group, then four to the control group, eight to the intervention group and the final four to the control group.

Allocation concealment was rated as a low risk of bias in seven studies because the concealment of allocation was likely due to the use of concealed envelopes or an independent or blinded investigator [37–39, 41, 42, 44, 49]. Three studies were rated as high risk of bias for allocation concealment [40, 46, 50]. One study used quasi-random assignment [49], and two studies allocated participants based on the order of recruitment [40, 46]. Eight studies had an unclear risk of bias because the method of allocation was not described [35, 36, 43, 45, 47, 48, 51, 52].

Four studies subjectively measured physical activity and were rated as a high risk of detection bias for blinding of outcome [35, 48–50]. The other studies used objectively measured physical activity outcomes and were rated as low risk of bias.

Attrition rates were reported in all studies. Four studies were rated as a high risk of bias on incomplete outcome data because of high drop-out [35, 36, 45, 47, 51]

One study was assessed as having a high risk of bias for selective outcome reporting because the outcome measures were not reported in detail in the paper [40].

Another potential bias was identified in two studies. In one study, 28% of the intervention completers used another weight loss intervention in addition to the intervention [35], and the second study was not registered in a trial registry [40].

Six studies were classified as having a low risk of bias [37–39, 41, 42, 44], six studies were classified as having an unclear risk of bias [43, 45, 47, 49, 51, 52] and six studies had a high risk of bias rating [35, 36, 40, 46, 48, 50].

### Publication Bias

We performed multiple meta-analyses since studies used different types of outcomes (i.e. moderate-to-vigorous physical activity or daily step count) and different methods of assessing physical activity (i.e. objectively and subjectively measured) and reported either change scores or outcome scores post-intervention and analysis including either studies with a low, unclear or high risk of bias or studies with only a low risk of bias. As a result, the number of studies was relatively low in each meta-analysis (< 10 studies), and we could not properly assess the funnel plots of publication bias [31].

### Effects of Wearables and Smartphone Applications on Physical Activity

#### Objectively Measured (Moderate-to-Vigorous) Physical Activity in Minutes Per Day (Change Scores)

**Fig. 4:**
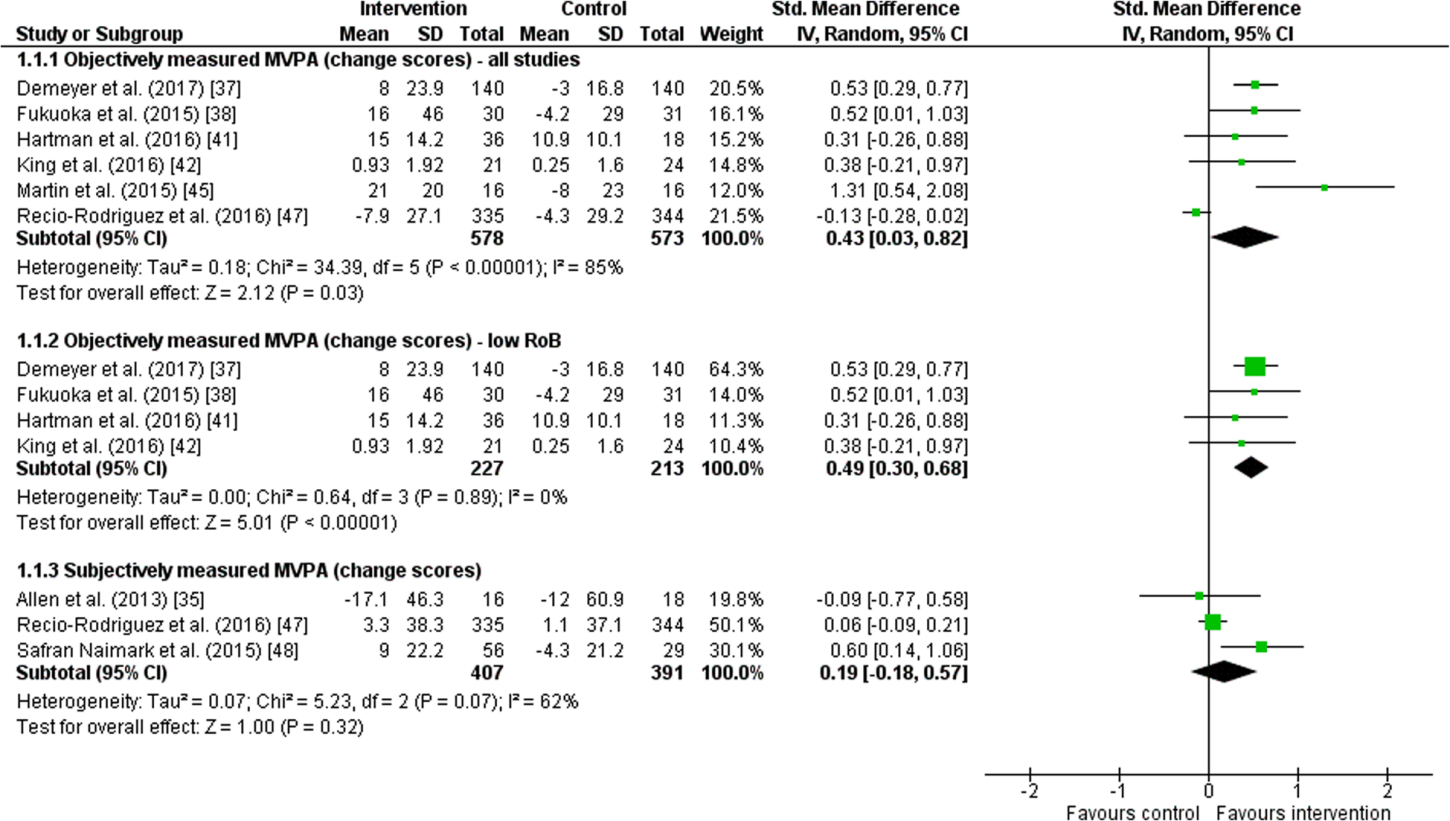
Forest plot of the effect of wearables and smartphone applications versus control on moderate-to-vigorous physical activity (MVPA) in minutes per day. *CI* confidence interval, *IV* inverse variance, *RoB* risk of bias, *SD* standard deviation, *Std* standardized

Six studies objectively measured physical activity and reported changes from baseline to post-intervention (Fig. 4). The random-effects meta-analysis showed an improvement in intervention groups compared to the control group; however, high statistical heterogeneity was detected (SMD = 0.43, 95% CI = 0.03 to 0.82; *I*^2^ = 85%). When excluding the two studies with an unclear or high risk of bias, a significant improvement was found in the intervention groups with no statistical heterogeneity (SMD = 0.49, 95% CI = 0.30 to 0.68; *I*^2^ = 0%).

#### Subjectively Measured (Moderate-to-Vigorous) Physical Activity in Minutes Per Day (Change Scores)

A change from baseline to post-intervention in subjectively measured physical activity was reported in three studies (Fig. 4). The random-effects meta-analysis showed no significant differences in physical activity level (SMD = 0.19, 95% CI = − 0.18 to 0.57; *I*^2^ = 62%).

#### Daily Step Count (Change Scores)

**Fig. 5:**
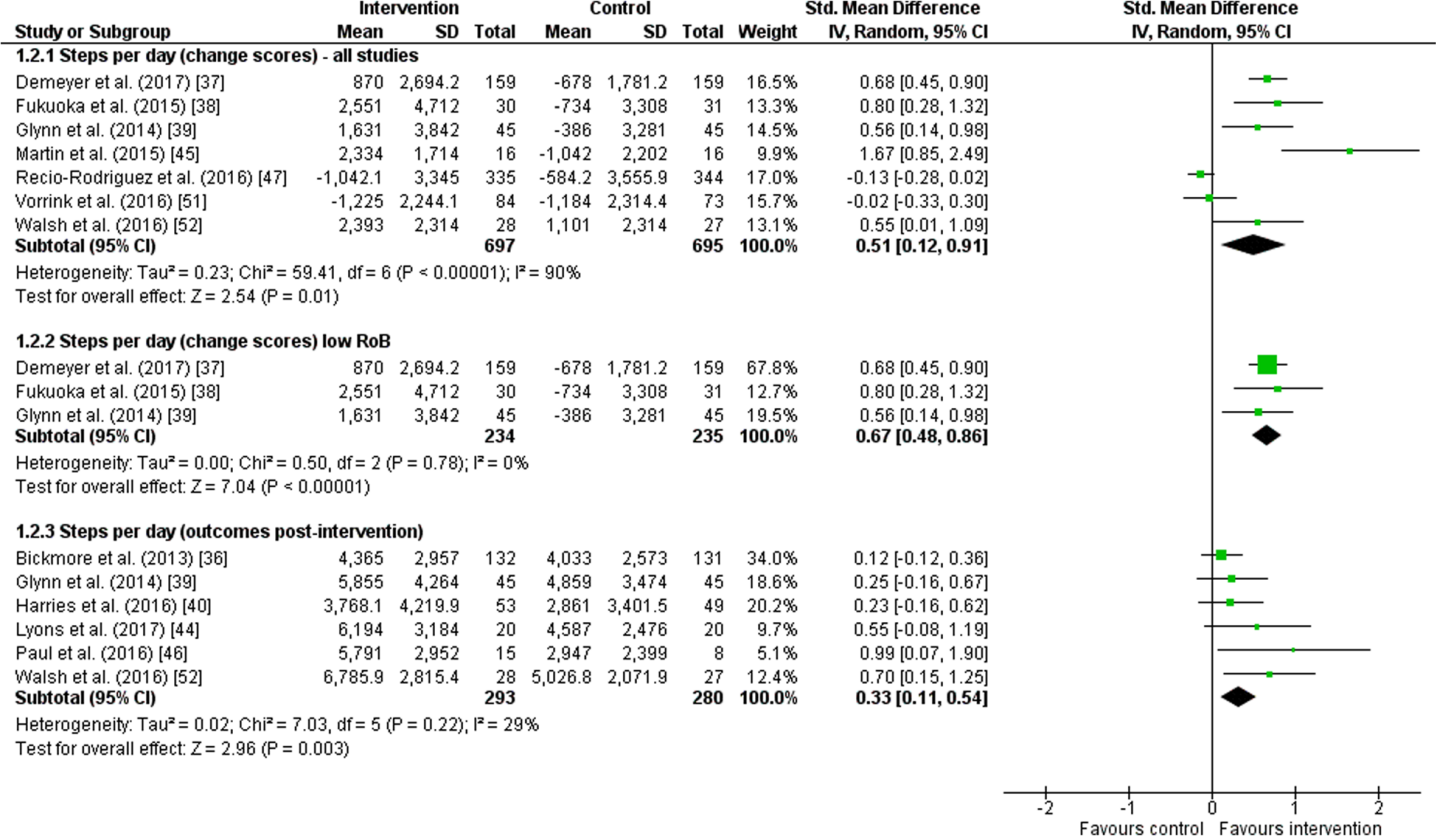
Forest plot of the effect of wearables and smartphone applications versus control on daily step count. *CI* confidence interval, *IV* inverse variance, *RoB* risk of bias, *SD* standard deviation, *Std* standardized

For change in daily step count from baseline to the end of the intervention as reported in seven studies, a significant improvement was found for intervention groups compared to control groups (SMD = 0.51, 95% CI = 0.12 to 0.91; *I*^2^ = 90%; Fig. 5). However, a high statistical heterogeneity was observed. When excluding the four studies with an unclear or high risk of bias, daily step count significantly improved in the intervention group compared to control with no statistical heterogeneity between studies (SMD = 0.67, 95% CI = 0.48 to 0.86; *I*^2^ = 0%).

#### Daily Step Count (Outcomes Post-intervention)

A random-effects meta-analysis including six studies demonstrated a significant improvement in daily step count post-intervention in intervention groups compared to control groups (SMD = 0.33, 95% CI = 0.11 to 0.54; *I*^2^ = 29%; Fig. 5). Moderate statistical heterogeneity was observed.

#### Studies with Other Outcomes

**Fig. 6:**
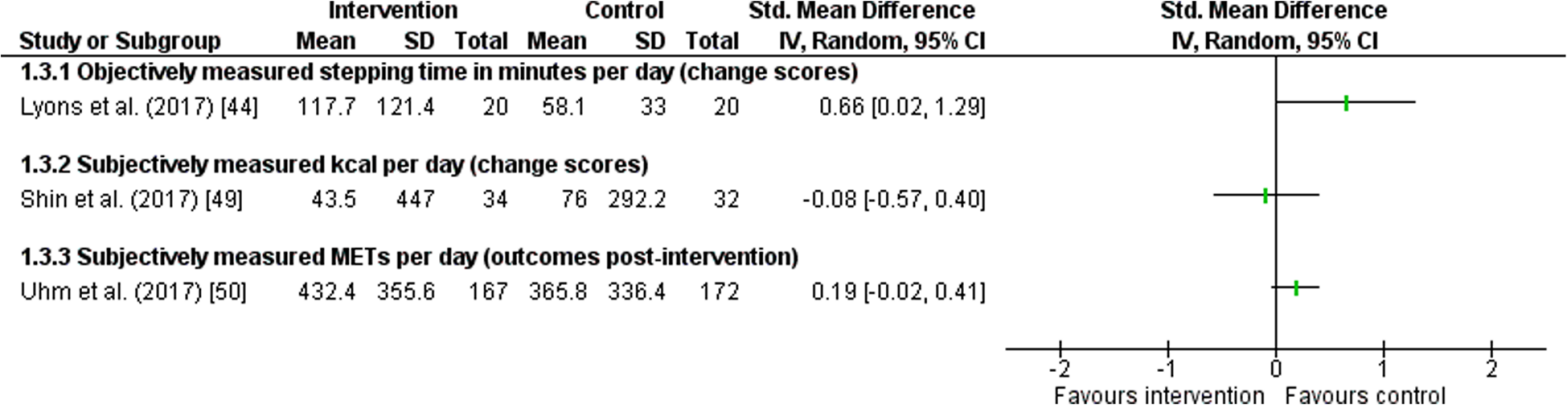
Forest plot of the effect of wearables and smartphone applications versus control on other outcomes. *CI* confidence interval, *IV* inverse variance, *MET* metabolic equivalent of task, *SD* standard deviation, *Std* standardized

Three studies reported other outcomes than (moderate-to-vigorous) physical activity in minutes or daily step count (Fig. 6) [38, 44, 45]. Reported outcomes were stepping time per day in minutes [39], kilocalories per day [43] and METs per day [45]. In all studies, no significant effect of the intervention was found in physical activity.

#### Subgroup Analysis

No differences were found between healthy [39, 47, 52] and diseased [37, 51] study populations (daily step count) and between interventions with an intervention duration shorter than 14 weeks [37, 42, 45, 47] and studies with a longer intervention duration [38, 41] (objectively measured physical activity).

## Discussion

This systematic review and meta-analysis showed that exercise interventions comprising wearables and smartphone applications were effective in promoting physical activity in adults. A moderate effect was found for objectively measured change in (moderate-to-vigorous) physical activity, and a moderate-to-large effect was found for change in daily step count. No significant effect was found for subjectively measured change in (moderate-to-vigorous) physical activity.

The results of this review are consistent with previous systematic reviews showing promising results of smartphone application in promoting physical activity [27, 28]; however, the results in these systematic reviews were based on a combination of randomized controlled trials and qualitative studies and no meta-analysis was performed. The current review included only randomized controlled trials and adds to the existing literature by conducting a meta-analysis. In addition, the above-mentioned systematic reviews targeted populations representative for the general population (i.e. patients with chronic disease were excluded). We included studies with different target populations, e.g. healthy adults, overweight or inactive adults, chronically diseased adults or older age groups.

Considerable statistical heterogeneity was detected between studies. Statistical heterogeneity between studies was removed when including only studies with a low risk of bias in the meta-analysis. Although also the number of studies was reduced in the ‘low risk of bias’ meta-analyses, analyses were still based on more than 400 subjects, implying sufficient power. Besides the differences in study quality, variation in study populations may explain the statistical heterogeneity. For example, previous research showed that more sedentary or inactive adults may benefit more from an intervention promoting physical activity than already active adults [53, 54]. In the same way, one might expect that patients with chronic disease may benefit more from an intervention promoting physical activity than healthy adults because of perceived barriers to exercise, e.g. disease-related fatigue. Subgroup analysis did not show a differential effect for healthy or diseased adults, probably because of the small number of studies included in this analysis.

Studies also varied in number and combination of included intervention components and behaviour change techniques. Promotion of physical activity through a smartphone application in combination with a wearable is in most studies accompanied by other intervention components, such as counselling sessions. Hence, no conclusions could be drawn about the isolated effect of wearables and smartphone applications on physical activity. Schoeppe et al. [28] showed that only offering a smartphone application is less effective than offering a smartphone application with additional intervention components. As a result, compared to only offering a wearable and smartphone application, intervention effects may be larger when the intervention is accompanied with other intervention components. Furthermore, goal setting is one of the most important behaviour change techniques to increase physical activity [53]. In most studies, physical activity goals were individualized, which may be more effective in promoting physical activity compared to general physical activity goals [55]. Other sources of heterogeneity might be variation in intervention duration and differences between control groups across studies, ranging from usual care to having a physical activity goal.

Variation in intervention characteristics (e.g. intervention components and duration) may be associated with the target population. Based on the results of this review, we could not draw conclusions on the optimal combination of intervention characteristics per target population. Future studies should therefore explore which (combination of) elements are effective in different populations by using for example factorial or Sequential Multiple Assignment Randomized Trial (SMART) designs.

It is still a challenge to reach healthy but physically inactive people and achieve a behaviour change [14–16]. Efforts have been undertaken to reach this population since physical inactivity is a key risk factor for developing different (chronic) diseases and premature death [5–11]. A promising strategy to foster motivation for physical activity is using the motivational power of games. For instance, mobile exergames as Pokémon GO, an augmented reality game in which players have to catch Pokémon (pocket monsters) that appear as virtual creatures in real places, appeared promising to reach a broad range of inactive people. First studies showed an increase in physical activity and more inactive populations were reached [56, 57]. However, physical activity decreased again within a few weeks indicating that people stopped playing [57, 58]. Future intervention studies should evaluate the potential added value of utilizing gaming strategies in smartphone applications in order to promote physical activity and to maintain change in physical activity level in the long term.

Most studies in this review focused on results directly post-intervention. Therefore, we could not obtain an insight into the sustainability of increased physical activity levels. Further research should include long-term follow-up assessments. Also, it would be interesting to obtain more insight into adherence to the use of wearables and smartphone applications and factors influencing adherence, e.g. personal preferences for apps and behaviour change techniques. Greater adherence may mediate the effect of the intervention on physical activity. With more information on sustainability, adherence and long-term effectiveness, wearables and smartphone apps could be designed that are most effective in promoting physical activity to optimize impact on public health. In this review, it was not possible to study the effectiveness of each behaviour change technique since they co-occur with other intervention characteristics and behaviour change techniques.

Lastly, we focused on the effect of physical activity. In future research, it would be interesting to focus on other outcomes as well, for example, quality of life and mood.

## Conclusions

To conclude, a physical activity intervention comprising a wearable and/or smartphone application is promising in promoting physical activity in adult populations. Most interventions also included other intervention components which might support the effects. Wearables and smartphone applications are likely to bring more opportunities in delivering tailored and effective interventions to increase physical activity.

## Appendix

### Search Strings

#### Search String PubMed (3161)

(((Mobile applications[MeSH] OR Cell Phones[MeSH] OR Actigraphy[MeSH] OR Application, Mobile[tiab] OR Applications, Mobile[tiab] OR Mobile Application[tiab] OR Mobile Apps[tiab] OR App, Mobile[tiab] OR Apps, Mobile[tiab] OR Mobile App[tiab] OR Application*[tiab] OR Apps[tiab] OR App[tiab] OR Application Software[tiab] OR Technological Appliance[tiab] OR Smartphone Application[tiab] OR Activity Tracker*[tiab] OR Wearable Technolog*[tiab] OR Wearable Device*[tiab] OR Smart Band*[tiab] OR Smart Watch*[tiab] OR Smartphone App*[tiab] OR Mobile Phone[tiab] OR Phone, Cell[tiab] OR Phones, Cell[tiab] OR Cellular Phone[tiab] OR Cellular Phones[tiab] OR Phone, Cellular[tiab] OR Phones, Cellular[tiab] OR Telephone, Cellular[tiab] OR Cellular Telephone[tiab] OR Cellular Telephones[tiab] OR Telephones, Cellular[tiab] OR Cell Phone[tiab] OR Smartphone[tiab] OR Smartphones[tiab] OR Smart Phones[tiab] OR Smart Phone[tiab] OR Mobile Phone[tiab] OR Mobile Phones[tiab] OR Phone, Mobile[tiab] OR Phones, Mobile[tiab] OR Mobile Telephone[tiab] OR Mobile Telephones[tiab] OR Telephone, Mobile[tiab] OR Telephones, Mobile[tiab] OR Accelorometr*[tiab])

AND

(Exercise[MeSH] OR Motor Activity[MeSH] OR Sedentary lifestyle[tiab] OR activities of daily liv*[tiab] OR Exercis*[tiab] OR Exercise, Physical[tiab] OR Exercises, Physical[tiab] OR Physical Exercise[tiab] OR Physical Exercises[tiab] OR Exercise, Aerobic[tiab] OR Aerobic Exercises[tiab] OR Exercises, Aerobic[tiab] OR Aerobic Exercise[tiab] OR Therapy, Exercise Therapy[tiab] OR Exercise Therapies[tiab] OR Therapies, Exercise[tiab] OR Physical fitness[tiab] OR Activity Behavio*[tiab] OR Activities, Motor[tiab] OR Activity, Motor[tiab] OR Motor Activities[tiab] OR Physical Activity[tiab] OR Activities, Physical[tiab] OR Activity, Physical[tiab] OR Physical Activities[tiab] OR Locomotor Activity[tiab] OR Activities, Locomotor[tiab] OR Activity, Locomotor[tiab] OR Locomotor Activities[tiab] OR Activity Level*[tiab] OR Activity Pattern[tiab] OR Exercise Behavio*[tiab] OR Exercise Behavio*[tiab] OR Exercise Pattern[tiab] OR Exercise Level[tiab] OR Physical Exercise[tiab] OR Physical Behaviour[tiab] OR Physical Behavior[tiab] OR Recreational Activity[tiab] OR Sport Behaviour[tiab] OR Sport Behavior[tiab] OR Fitness[tiab])))

AND

2015/09: 2017/07[dp]

#### Search String Embase (8557)

(‘mobile application’/exp. OR ‘mobile phone’/exp. OR ‘actimetry’/exp. OR ‘application, mobile’:ti,ab OR ‘applications, mobile’:ti,ab OR ‘mobile application’:ti,ab OR ‘mobile apps’:ti,ab OR ‘app, mobile’:ti,ab OR ‘apps, mobile’:ti,ab OR ‘mobile app’:ti,ab OR ‘application*’:ti,ab OR ‘apps’:ti,ab OR ‘app’:ti,ab OR ‘application software’:ti,ab OR ‘technological appliance’:ti,ab OR ‘smartphone application’:ti,ab OR ‘activity tracker*’:ti,ab OR ‘wearable technolog*’:ti,ab OR ‘wearable device*’:ti,ab OR ‘smart band*’:ti,ab OR ‘smart watch*’:ti,ab OR ‘smartphone app’:ti,ab OR ‘mobile phone’:ti,ab OR ‘phone, cell’:ti,ab OR ‘phones, cell’:ti,ab OR ‘cellular phone’:ti,ab OR ‘cellular phones’:ti,ab OR ‘phone, cellular’:ti,ab OR ‘telephone, cellular’:ti,ab OR ‘cellular telephone’:ti,ab OR ‘cellular telephones’:ti,ab OR ‘telephones, cellular’:ti,ab OR ‘cell phone’:ti,ab OR ‘smartphone’:ti,ab OR ‘smartphones’:ti,ab OR ‘smart phone*’:ti,ab OR ‘mobile phone*’:ti,ab OR ‘mobile telephone*’:ti,ab OR ‘telephone*, mobile’:ti,ab OR ‘accelorometr*’:ti,ab)

AND

(‘motor activity’/exp. OR ‘physical activity’/exp. OR ‘exercise’/exp. OR ‘sedentary lifestyle’:ti,ab OR ‘activities of daily liv*’:ti,ab OR ‘exercis*’:ti,ab OR ‘exercise*, physical’:ti,ab OR ‘physical exercise*’:ti,ab OR ‘exercise*, aerobic’:ti,ab OR ‘aerobic exercise*’:ti,ab OR ‘therap*, exercise’:ti,ab OR ‘exercise therap*’:ti,ab OR ‘physical fitness’:ti,ab OR ‘activity behavio*’:ti,ab OR ‘activit*, motor’:ti,ab OR ‘motor activit*’:ti,ab OR ‘physical activit*’:ti,ab OR ‘activit*, physical’:ti,ab OR ‘locomotor activit*’:ti,ab OR ‘activit*, locomotor’:ti,ab OR ‘activity level*’:ti,ab OR ‘activity pattern’:ti,ab OR ‘exercise behavio*’:ti,ab OR ‘exercise pattern’:ti,ab OR ‘exercise level’:ti,ab OR ‘physical exercise’:ti,ab OR ‘physical behavi*’:ti,ab OR ‘recreational activity’:ti,ab OR ‘sport behavio*’:ti,ab OR ‘fitness’:ti,ab)

AND

[1-9-2015]/sd NOT [31-7-2017]/sd

#### Search String Cochrane (980)

([mh “Mobile Applications”] or [mh “Cell Phones”] or [mh “Actigraphy”] or (Application, Mobile or Applications, Mobile or Mobile Application or Mobile Apps or App, Mobile or Apps, Mobile or Mobile App or Application* or Apps or App or Application Software or Technological Appliance or Smartphone Application or Activity Tracker* or Wearable Technolog* or Wearable Device* or Smart Band* or Smart Watch* or Smartphone App* or Mobile Phone or Phone, Cell or Phones, Cell or Cellular Phone or Cellular Phones or Phone, Cellular or Phones, Cellular or Telephone, Cellular or Cellular Telephone or Cellular Telephones or Telephones, Cellular or Cell Phone or Smartphone or Smartphones or Smart Phones or Smart Phone or Mobile Phone or Mobile Phones or Phone, Mobile or Phones, Mobile or Mobile Telephone or Mobile Telephones or Telephone, Mobile or Telephones, Mobile or Accelorometr*):ti,ab,kw)

AND

([mh “Motor Activity”] or [mh “Exercise”] or (Sedentary lifestyle or activities of daily liv* or exercis* or Exercise, Physical or Exercises, Physical or Physical Exercise or Physical Exercises or Exercise, Aerobic or Aerobic Exercises or Exercises, Aerobic or Aerobic Exercise or Therapy, Exercise Therapy or Exercise Therapies or Therapies, Exercise or Physical fitness or Activity Behavio* or Activities, Motor or Activity, Motor or Motor Activities or Physical Activity or Activities, Physical or Activity, Physical or Physical Activities or Locomotor Activity or Activities, Locomotor or Activity, Locomotor or Locomotor Activities or Activity Level* or Activity Pattern or Exercise Behavio* or Exercise Behavio* or Exercise Pattern or Exercise Level or Physical Exercise or Physical Behaviour or Physical Behavior or Recreational Activity or Sport Behaviour or Sport Behavior or Fitness): ti,ab,kw)

Limit: Publication Year from 2015 to 2017 in Trials.

## Abbreviations

CI: Confidence interval
METs: Metabolic equivalent of task
MPA: Moderate physical activity
MVPA: Moderate-to-vigorous physical activity
PA: Physical activity
RCT: Randomized controlled trial
RoB: Risk of bias
SMD: Standardized mean difference

## References

[1] Andersen LB, Mota J, Di Pietro L (2016). Update on the global pandemic of physical inactivity. Lancet.

[2] Hallal PC, Andersen LB, Bull FC, Guthold R, Haskell W, Ekelund U (2012). Global physical activity levels: surveillance progress, pitfalls, and prospects. Lancet.

[3] Kohl HW, Craig CL, Lambert EV (2012). The pandemic of physical inactivity: global action for public health. Lancet.

[4] Tremblay MS, Aubert S, Barnes JD (2017). Sedentary Behavior Research Network (SBRN) - terminology consensus project process and outcome. Int J Behav Nutr Phys Act.

[5] Ding D, Lawson KD, Kolbe-Alexander TL (2016). The economic burden of physical inactivity: a global analysis of major non-communicable diseases. Lancet.

[6] Lee IM, Shiroma EJ, Lobelo F, Puska P, Blair SN, Katzmarzyk PT (2012). Effect of physical inactivity on major non-communicable diseases worldwide: an analysis of burden of disease and life expectancy. Lancet.

[7] Kyu HH, Bachman VF, Alexander LT (2016). Physical activity and risk of breast cancer, colon cancer, diabetes, ischemic heart disease, and ischemic stroke events: systematic review and dose-response meta-analysis for the Global Burden of Disease Study 2013. BMJ.

[8] Biswas A, Oh PI, Faulkner GE (2015). Sedentary time and its association with risk for disease incidence, mortality, and hospitalization in adults: a systematic review and meta-analysis. Ann Intern Med.

[9] Organisation WH (2015). Global status report on noncommunicable diseases 2014.

[10] Proper KI, Singh AS, van Mechelen W, Chinapaw MJ (2011). Sedentary behaviors and health outcomes among adults: a systematic review of prospective studies. Am J Prev Med.

[11] Wilmot EG, Edwardson CL, Achana FA (2012). Sedentary time in adults and the association with diabetes, cardiovascular disease and death: systematic review and meta-analysis. Diabetologia.

[12] Arem H, Moore SC, Patel A (2015). Leisure time physical activity and mortality: a detailed pooled analysis of the dose-response relationship. JAMA Intern Med.

[13] Ekelund U, Steene-Johannessen J, Brown WJ (2016). Does physical activity attenuate, or even eliminate, the detrimental association of sitting time with mortality? A harmonised meta-analysis of data from more than 1 million men and women. Lancet.

[14] McDermott MS, Oliver M, Iverson D, Sharma R (2016). Effective techniques for changing physical activity and healthy eating intentions and behaviour: a systematic review and meta-analysis. Br J Health Psychol.

[15] Murray JM, Brennan SF, French DP, Patterson CC, Kee F, Hunter RF (2017). Effectiveness of physical activity interventions in achieving behaviour change maintenance in young and middle aged adults: a systematic review and meta-analysis. Soc Sci Med.

[16] Samdal GB, Eide GE, Barth T, Williams G, Meland E (2017). Effective behaviour change techniques for physical activity and healthy eating in overweight and obese adults; systematic review and meta-regression analyses. Int J Behav Nutr Phys Act.

[17] Michie S, Abraham C, Whittington C, McAteer J, Gupta S (2009). Effective techniques in healthy eating and physical activity interventions: a meta-regression. Health Psychol.

[18] Direito A, Pfaeffli Dale L, Shields E, Dobson R, Whittaker R, Maddison R (2014). Do physical activity and dietary smartphone applications incorporate evidence-based behaviour change techniques?. BMC Public Health.

[19] Kang M, Marshall SJ, Barreira TV, Lee JO (2009). Effect of pedometer-based physical activity interventions: a meta-analysis. Res Q Exerc Sport.

[20] Webb TL, Joseph J, Yardley L, Michie S (2010). Using the internet to promote health behavior change: a systematic review and meta-analysis of the impact of theoretical basis, use of behavior change techniques, and mode of delivery on efficacy. J Med Internet Res.

[21] Davies CA, Spence JC, Vandelanotte C, Caperchione CM, Mummery WK (2012). Meta-analysis of internet-delivered interventions to increase physical activity levels. Int J Behav Nutr Phys Act.

[22] Sanders JP, Loveday A, Pearson N (2016). Devices for self-monitoring sedentary time or physical activity: a scoping review. J Med Internet Res.

[23] Number of smartphone users worldwide from 2014 to 2020 (in billions). Accessed Apr 2018. Available from: https://www.statista.com/statistics/330695/number-of-smartphone-users-worldwide/

[24] Android Statistics Top categories. Accessed Apr 2018. Available from: http://www.appbrain.com/stats/android-market-app-categories.

[25] Rabin C, Bock B (2011). Desired features of smartphone applications promoting physical activity. Telemed J E Health.

[26] Middelweerd A, Mollee JS, van der Wal C, Brug J, te Velde SJ (2014). Apps to promote physical activity among adults: a review and content analysis. Int J Behav Nutr Phys Act.

[27] Coughlin SS, Whitehead M, Sheats JQ, Mastromonico J, Smith SA (2016). Review of smartphone applications for promoting physical activity. Jacobs J Community Med.

[28] Schoeppe S, Alley S, Van Lippevelde W (2016). Efficacy of interventions that use apps to improve diet, physical activity and sedentary behaviour: a systematic review. Int J Behav Nutr Phys Act.

[29] Moher D, Liberati A, Tetzlaff J, Altman DG, Group TP (2009). Preferred Reporting Items for Systematic Reviews and Meta-analyses: the PRISMA statement. PLoS Med.

[30] Higgins JPT, Green S. Cochrane Handbook for Systematic Reviews of Interventions Version 5.1.0 [updated March 2011]. The Cochrane Collaboration; 2011. Available from http://handbook.cochrane.org.

[31] Vanhees L, Lefevre J, Philippaerts R (2005). How to assess physical activity? How to assess physical fitness?. Eur J Prev Cardiol.

[32] Review Manager (RevMan) [Computer program]. Version 5.3. Copenhagen: The Nordic Cochrane Centre The Cochrane Collaboration; 2014. https://community.cochrane.org/help/tools-and-software/revman-5.

[33] Cohen J (1988). Statistical power analysis for the behavioral sciences.

[34] Higgins JP, Thompson SG, Deeks JJ, Altman DG (2003). Measuring inconsistency in meta-analyses. BMJ.

[35] Allen JK, Stephens J, Dennison Himmelfarb CR, Stewart KJ, Hauck S (2013). Randomized controlled pilot study testing use of smartphone technology for obesity treatment. J Obes.

[36] Bickmore TW, Silliman RA, Nelson K (2013). A randomized controlled trial of an automated exercise coach for older adults. J Am Geriatr Soc.

[37] Demeyer H, Louvaris Z, Frei A (2017). Physical activity is increased by a 12-week semiautomated telecoaching programme in patients with COPD: a multicentre randomised controlled trial. Thorax.

[38] Fukuoka Y, Gay CL, Joiner KL, Vittinghoff E (2015). A novel diabetes prevention intervention using a mobile app: a randomized controlled trial with overweight adults at risk. Am J Prev Med.

[39] Glynn LG, Hayes PS, Casey M (2014). Effectiveness of a smartphone application to promote physical activity in primary care: the SMART MOVE randomised controlled trial. Br J Gen Pract.

[40] Harries T, Eslambolchilar P, Rettie R, Stride C, Walton S, van Woerden HC (2016). Effectiveness of a smartphone app in increasing physical activity amongst male adults: a randomised controlled trial. BMC Public Health.

[41] Hartman SJ, Nelson SH, Cadmus-Bertram LA, Patterson RE, Parker BA, Pierce JP (2016). Technology and phone based weight loss intervention: pilot RCT in women at elevated breast cancer risk. Am J Prev Med.

[42] King AC, Hekler EB, Grieco LA (2016). Effects of three motivationally targeted mobile device applications on initial physical activity and sedentary behavior change in midlife and older adults: a randomized trial. PLoS One.

[43] Li LC, Sayre EC, Xie H, Clayton C, Feehan LM (2017). A community-based physical activity counselling program for people with knee osteoarthritis: feasibility and preliminary efficacy of the track-OA study. JMIR Mhealth Uhealth.

[44] Lyons EJ, Swartz MC, Lewis ZH, Martinez E, Jennings K (2017). Feasibility and acceptability of a wearable technology physical activity intervention with telephone counseling for mid-aged and older adults: a randomized controlled pilot trial. JMIR Mhealth Uhealth.

[45] Martin SS, Feldman DI, Blumenthal RS (2015). MActive: a randomized clinical trial of an automated mHealth intervention for physical activity promotion. J Am Heart Assoc.

[46] Paul L, Wyke S, Brewster S (2016). Increasing physical activity in stroke survivors using STARFISH, an interactive mobile phone application: a pilot study. Top Stroke Rehabil.

[47] Recio-Rodriguez JI, Agudo-Conde C, Martin-Cantera C (2016). Short-term effectiveness of a mobile phone app for increasing physical activity and adherence to the Mediterranean diet in primary care: a randomized controlled trial (EVIDENT II study). J Med Internet Res.

[48] Safran Naimark J, Madar Z, Shahar DR (2015). The impact of a web-based app (eBalance) in promoting healthy lifestyles: randomized controlled trial. J Med Internet Res.

[49] Shin DW, Yun JM, Shin JH (2017). Enhancing physical activity and reducing obesity through smartcare and financial incentives: a pilot randomized trial. Obesity (Silver Spring).

[50] Uhm KE, Yoo JS, Chung SH (2017). Effects of exercise intervention in breast cancer patients: is mobile health (mHealth) with pedometer more effective than conventional program using brochure?. Breast Cancer Res Treat.

[51] Vorrink SN, Kort HS, Troosters T, Zanen P, Lammers JJ (2016). Efficacy of an mHealth intervention to stimulate physical activity in COPD patients after pulmonary rehabilitation. Eur Respir J.

[52] Walsh JC, Corbett T, Hogan M, Duggan J, McNamara A (2016). An mHealth intervention using a smartphone app to increase walking behavior in young adults: a pilot study. JMIR Mhealth Uhealth.

[53] Bravata DM, Smith-Spangler C, Sundaram V (2007). Using pedometers to increase physical activity and improve health: a systematic review. JAMA.

[54] Chan CB, Ryan DA, Tudor-Locke C (2004). Health benefits of a pedometer-based physical activity intervention in sedentary workers. Prev Med.

[55] Locke E, Latham G (2006). New directions in goal-setting theory. Curr Dir Psychol Sci.

[56] Althoff T, White RW, Horvitz E (2016). Influence of Pokemon Go on physical activity: study and implications. J Med Internet Res.

[57] Howe KB, Suharlim C, Ueda P, Howe D, Kawachi I, Rimm EB (2016). Gotta catch’em all! Pokemon GO and physical activity among young adults: difference in differences study. BMJ.

[58] Rasche P, Schlomann A, Mertens A (2017). Who is still playing Pokemon Go? A web-based survey. JMIR Serious Games.

